# Mastitis

**Published:** 1889-07

**Authors:** Wm. Jefferson Guernsey


### MASTITIS.

Wm. Jefferson Guernsey, M. D.

As mastitis rarely occurs except early in lactation, the care of the breast cannot be too soon observed after confinement.

First, last, and all the time see that there is ample protection from the air. Warmth is a wonderful factor in promoting glandular health and activity, and nothing answers the purpose better than a piece of soft flannel, secured at either upper corner near the shoulders and allowed to lay over both mammæ, and lifted up, (not let down nor removed), while suckling the child.

It is an important point also to have the child commence nursing as early as convenient to the mother. Chilling of other parts of the body, especially of the hands, is a frequent cause of trouble, and checked perspiration, mental irritation, and malnutrition are to be avoided at any time.

So much for general attention to health, yet in spite of gross carelessness, of poor food, or a combination of circumstances the homœopathic prescription will always prevent suppuration if taken in time, and there is little satisfaction to the physician in treating any abscess through suppuration to resolution, for he knows that it is a catastrophe that should indeed have been averted.

Medicines will do much to hasten suppuration when it is inevitable, and as to local measures I can see no reasonable objection to poulticing. Holding a basin of hot water under the breast, and sponging the upper part of the gland from it will be beneficial, especially if followed by warm wrappings, of which raw wool is the best. I believe that the breast should *never* be lanced. The opening which nature makes is smaller than that occasioned by the knife, is always made at the point nearest the surface, and a recurrence of the trouble is less likely from a natural evacuation of the pus, especially if a careful selection of the homœopathic remedy has been made.

As *Lac-can.* and *Phytol.* are far ahead of any other remedies in aborting this trouble, a comparison may be of service.

| LAC-CAN. | PHYTOL. |
|---|---|
| Affects one breast as much as the other; as *Phytol.* acts particularly on the right and Lac-can. *either*, it may be given preference to the *left*. If there has been soreness or pain alternating from one breast to the other, or migratory trouble of any sort about the patient it should be used. | *Right* breast. |
| Much soreness, fullness, and pain, but not so much inflammation, although this latter should not rule it out of consideration. | Inflammation marked with soreness, fullness, and pain. |
| Very much worse from least jar; has to support the breast in walking about, especially on going up or downstairs. Even worse from inspiration. | Not so pronounced. |
| Induration in small lumps like marbles. Considering the fact that its membranous exudation in the throat is in small specks, I have (on the rule of similars) marked this "nodulated breast" high under Lac-can. | Same in lesser degree, but it has cured for me many cases of a *single* stony induration. |
| Markedly worse *toward evening*—and EVENING. | Worse after midnight; better in afternoon. |

